# Dietary Eugenol Nanoemulsion Potentiated Performance of Broiler Chickens: Orchestration of Digestive Enzymes, Intestinal Barrier Functions and Cytokines Related Gene Expression With a Consequence of Attenuating the Severity of *E. coli* O78 Infection

**DOI:** 10.3389/fvets.2022.847580

**Published:** 2022-06-23

**Authors:** Doaa Ibrahim, Fatma Eldemery, Aya Sh. Metwally, Ehab M. Abd-Allah, Dalia T. Mohamed, Tamer Ahmed Ismail, Thoria A. Hamed, Gehan M. Al Sadik, Ahmed N. F. Neamat-Allah, Marwa I. Abd El-Hamid

**Affiliations:** ^1^Department of Nutrition and Clinical Nutrition, Faculty of Veterinary Medicine, Zagazig University, Zagazig, Egypt; ^2^Department of Hygiene and Zoonoses, Faculty of Veterinary Medicine, Mansoura University, Mansoura, Egypt; ^3^Department of Pharmacology, Faculty of Veterinary Medicine, Aswan University, Aswan, Egypt; ^4^Veterinary Educational Hospital, Faculty of Veterinary Medicine, Zagazig University, Zagazig, Egypt; ^5^Department of Pathology and Clinical Pathology, Zagazig Branch, Agriculture Research Center, Animal Health Research Institute, Zagazig, Egypt; ^6^Department of Clinical Laboratory Sciences, Turabah University College, Taif University, Taif, Saudi Arabia; ^7^Department of Biochemistry, Zagazig Branch, Agriculture Research Center, Animal Health Research Institute, Zagazig, Egypt; ^8^Department of Bacteriology, Zagazig Branch, Agriculture Research Center, Animal Health Research Institute, Zagazig, Egypt; ^9^Department of Clinical Pathology, Faculty of Veterinary Medicine, Zagazig University, Zagazig, Egypt; ^10^Department of Microbiology, Faculty of Veterinary Medicine, Zagazig University, Zagazig, Egypt

**Keywords:** broiler chickens, eugenol nanoemulsion, performance, immunity, barrier function, APEC O78, virulence gene expression

## Abstract

Recently, the use of essential oils (EOs) or their bioactive compounds encapsulated by nanoparticles as alternative supplements for in-feed antimicrobials is gaining attention, especially in organic poultry production. Focusing on eugenol, its incorporation into the nanoformulation is a novel strategy to improve its stability and bioavailability and thus augment its growth-boosting and antimicrobial activities. Therefore, we explored eugenol nanoemulsion activities in modulating growth, digestive and gut barrier functions, immunity, cecal microbiota, and broilers response to avian pathogenic *E. coli* challenge (APEC) O78. A total of 1,000 one-day-old broiler chicks were allocated into five groups; negative control (NC, fed basal diet), positive control (PC), and 100, 250, and 400 mg/kg eugenol nanoemulsion supplemented groups. All groups except NC were challenged with APEC O78 at 14 days of age. The results showed that birds fed eugenol nanoemulsion displayed higher BWG, FI, and survivability and most improved FCR over the whole rearing period. Birds fed 400 mg/kg of eugenol nanoemulsion sustained a higher growth rate (24% vs. PC) after infection. Likely, the expression of digestive enzymes' genes (*AMY2A, CCK, CELA1*, and *PNLIP*) was more prominently upregulated and unaffected by APEC O78 challenge in the group fed eugenol nanoemulsion at the level of 400 mg/kg. Enhanced gut barrier integrity was sustained post-challenge in the group supplemented with higher levels of eugenol nanoemulsion as evidenced by the overexpression of cathelicidins-2, β-defensin-1, *MUC*-2, *JAM*-2, occludin, *CLDN*-1, and *FABP*-2 genes. A distinct modulatory effect of dietary eugenol nanoemulsion was observed on cytokine genes (IL-1β, TNF-α, *IL*-6, *IL*-8, and *IL*-10) expression with a prominent reduction in the excessive inflammatory reactions post-challenge. Supplementing eugenol nanoemulsion increased the relative cecal abundance of *Lactobacillus* species and reduced *Enterobacteriaceae* and *Bacteriods* counts. Notably, a prominent reduction in APEC O78 loads with downregulation of *papC, iroN, iutA*, and *iss* virulence genes and detrimental modifications in *E. coli* morphological features were noticed in the 400 mg/kg eugenol nanoemulsion group at the 3rd-week post-challenge. Collectively, we recommend the use of eugenol nanoemulsion as a prospective targeted delivery approach for achieving maximum broilers growth and protection against APEC O78 infection.

## Introduction

The overuse of in-feed antibiotics in the poultry industry had increased the emergence of antibiotic-resistant bacterial strains (1, 2) and drug residues in meat posing a risk to public health and environment (3). Thus, regulations concerning the use of antibiotic growth promoters or increasing consumer necessity for poultry products free from antibiotics have increased the pursuit of alternative products. Nowadays, there is a renewed interest in developing safe and eco-friendly immunomodulating, antioxidant, and antibacterial alternative natural agents (4). In particular, phytogenic feed additives or their compounds, i.e., essential oils (EOs) have been displayed to modify gut signaling molecules (5), gut (6) and luminal (7) microbiota composition, gut integrity (8–10) and pro- and anti-inflammatory cytokines expression (11–13). Other features of these additives include their capability for increasing the growth performance, digestive functions, or expression of digestive enzyme related genes (14–17) and to enhance the meat quality in broiler chickens (18–20). Among many potentially bioactive compounds from plant extracts, eugenol (4-allyl-2-methoxyphenol), a component of clove oil (21), has revealed positive potential impacts on growth performance and intestinal health (22) due to its antioxidant, antibacterial, and anti-inflammatory properties (23). It possesses antibacterial efficacy against Gram-positive and Gram-negative bacteria (24). Many studies have provided direct evidence about its antibacterial activity, which is closely associated with its ability for permeabilization of the bacterial cell membrane, destroying membrane integrity and facilitating the entry of eugenol into the cytoplasm, which finally interacts with proteins and enzymes leading to the outflow of intracellular elements (23, 25, 26).

*Escherichia coli* (*E. coli*), the focus of this study, is a common member of the gut microbial community, while avian pathogenic *E. coli* (APEC) strains, especially O78 causes colibacillosis with a consequence of poorer bird performance and major economic losses due to higher morbidity and mortality rates (27). About 10–15% of avian gastrointestinal tract (GIT) coliforms have been demonstrated to belong to the potentially pathogenic APEC serogroups (28). Consistently, virulent and avirulent *E. coli* are shown to colonize and efficiently persist in the GIT with extra-intestinal translocation arising only under the existence of stressors (29). Strikingly, the intestinal location of APEC offers a good opportunity for spread into the environment and transmission through feces with a capability for efficient persistence in the dry environment. The dust in avian houses may have up to 10^6^ colony-forming units (CFU) of *E. coli* per gram (28). In spite of advances in poultry production systems over recent years, APEC still pose a challenge to poultry farmers and threaten food security at a time of growing global demand. This could be attributed to the development of free-range production systems that could increase the incidence of colibacillosis due to over-exposure of broiler chickens to environmental bacterial pathogens, injury, and stress (30). The use of antibiotics remains important in treating such bacterial infections; however, non-antibiotic strategies are essential for APEC diseases, for which antibiotic therapies are not optional. The merits of nanotechnology have combined to introduce a source of new marketable products in the research and field. Concerning technical stability, nanosystems have the benefits of protecting active ingredients against inactivation and degradation besides incorporation of ingredients with dissimilar polarity to trigger extended release and/or target a specific tissue (31). Among these nanocarriers, nanoemulsions are considered suitable carriers for active ingredients of EOs owing to their easy preparation, high surface area and stability, low cost of production, and the potential of production on large industrial scale for pharmacological and biological applications (32). In this context, the precise mode of actions of eugenol nanoemulsion beyond its promising effects with a special reference in controlling APEC and restoring broilers productivity in antibiotics-free system need forceful investigation. Hence, our experimental study evaluated, for the first time, the efficacy of eugenol nanoemulsion on the growth performance, gut barrier and digestive functions, immune response, and cecal microbiology at the molecular levels. Since, there were no data regarding the use of eugenol nanoemulsion as a potential candidate against APEC, we elucidated its optimistic role in the reluctance of colisepticemia experimentally induced in broilers focusing on the morphological alterations of APEC and modulation of its virulence gene expression as well.

## Materials and Methods

### Ethical Approval

The experiment procedures were approved by the University Strategies for the Care of Experimental Animals and they have been certified by the ZU-IACUC Board of the Faculty of Veterinary Medicine, Zagazig University, Egypt.

### Preparation and Characterization of Eugenol Nanoemulsion

Eugenol (W246719-1KG-K, Mol. Wt. 164.20), medium viscosity sodium alginate (Code A-2033), and polyoxyethylene (20) sorbitan monooleate were purchased from Sigma-Aldrich (Saint Louis, MO, United States). For oil phase preparation, sodium alginate was dissolved in hot distilled water at 70°C under continuous stirring until full solubility. A primary emulsion was prepared by blending the aqueous sodium alginate solution and eugenol essential oil (1% v/v) plus Tween 80 (1% v/v) as nonionic surfactant using a digital Ultra-Turrax disperser (IKA, Germany) for 2 min at 3,400 rpm. Ultrapure water was used as a solvent during all preparations. Afterward, the coarse emulsion was homogenized at 10,000 rpm for 10 min until the formation of nanoemulsion solution. This mixture was then sonicated through a Sonopuls HD 2200 ultrasonicator (Bandelin Berlin, Germany) at 700 W for 10 min.

**Figure 1:**
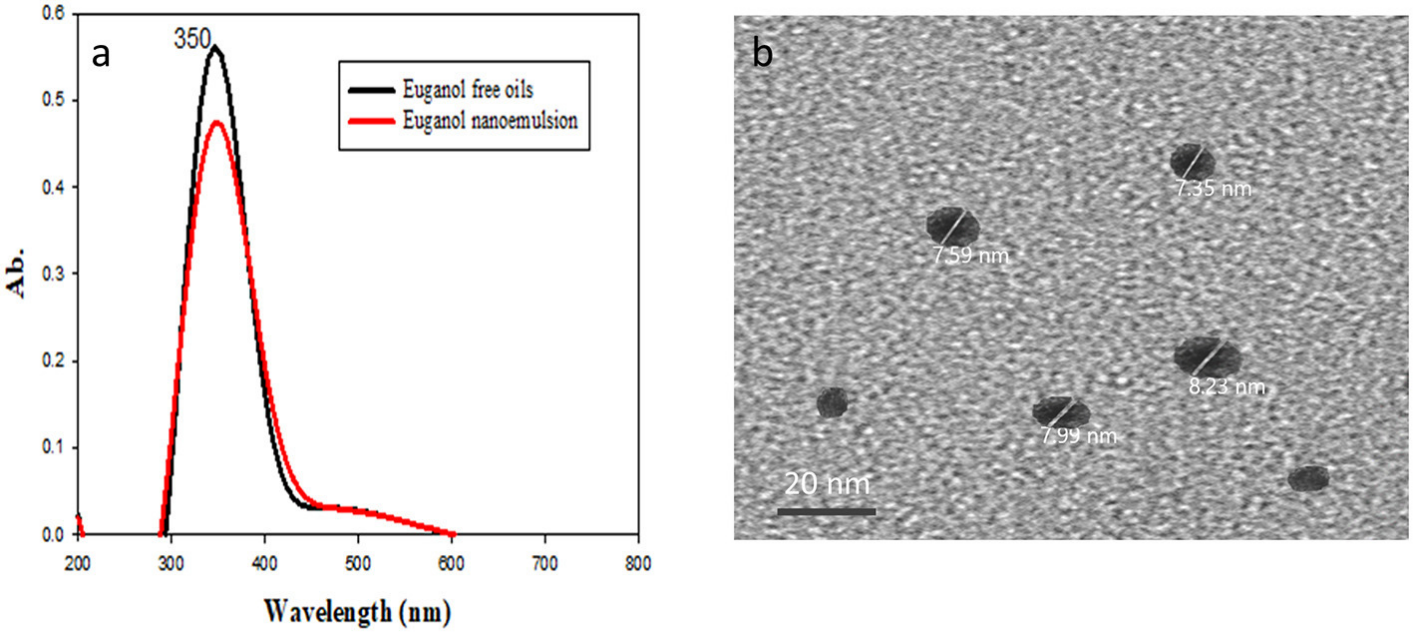
Fourier transform infrared (FTIR) spectroscopy **(a)** and transmission electron microscopy (TEM, **b**) of eugenol nanoemulsion.

Characterization of the prepared eugenol nanoemulsion was carried out by Fourier transform infrared (FTIR) spectroscopy (Figure 1a) at Radioactive Isotopes and Generators, Atomic Energy Authority, Egypt) and transmission electron microscopy (TEM, Figure 1b) at the National Center for Radiation Research and Technology, Egyptian Atomic Energy Authority, Cairo, Egypt.

### Experimental Chicks and Design

This experimental trial was carried out on 1,000 one-day-old broiler chicks (Ross 308), which were purchased from the Zagazig Poultry House hatchery. Upon arrival, they were separately weighed (initial weight = 44 ± 1.2 g), stayed in a deep litter poultry house for 35 days with an initial temperature of 34°C, which was slowly reduced to 24°C (±2°C) at the end of the 3rd week and kept in completely hygienic conditions. The chicks were divided into five groups (200 /group with 10 replicates/ 20 chicks each). The first two bird groups were served as negative control (NC, fed a control diet without eugenol nanoemulsion and were not challenged) and positive control (PC, fed a control diet without eugenol nanoemulsion and were challenged at 14 days of age with APEC O78 strain). The birds in the other three treatment groups were fed eugenol nanoemulsion with concentrations of 100, 250, and 400 mg/kg diet, respectively, and challenged with APEC O78. All birds were permitted admission to feed and water *ad libitum* during the 35-days experimental period. The control diet for starter (1–10 days), grower (11–20 days), and finisher (21–35 days) periods was formulated to meet Aviagen recommendations (33) as presented in Table 1. The chemical analyses of all feed ingredients and diets were done according to the standard methods recommended by the Association of Official Analytical Chemists (34).

**Table 1:** The ingredients and nutrient contents of basal diet.

**Ingredient, %**	**Starter (1–10 days)**	**Grower** **(11–20 days)**	**Finisher (21–35 days)**
Yellow corn	59.00	61.50	65.50
Soybean meal, 48%	34.40	30.80	25.80
Soybean oil	1.80	3.00	4.00
Calcium carbonate	1.20	1.20	1.20
Calcium diphasic phosphate	1.50	1.50	1.50
Common salt	0.30	0.30	0.3
Premix*	0.90	0.90	0.9
L-Lysine HCL, 78%	0.35	0.3	0.3
DL-Methionine, 99%	0.25	0.2	0.2
Choline chloride	0.20	0.20	0.20
Anti-mycotoxin	0.10	0.10	0.10
Calculated composition			
Metabolizable energy (Kcal/Kg)	3,106	3,103	3,200
Crude protein, %	23.01	21.5	19.50
Ether extract, %	4.33	5.6	6.62
Crude fiber, %	2.63	2.56	2.46
Calcium, %	1.20	1.19	1.17
Available phosphorous, %	0.53	0.50	0.48
Lysine, %	1.45	1.29	1.16
Methionine, %	0.58	0.51	0.49

^*^Vitamin premix supplied per kilogram of diet: retinol, 10.000 IU; tocopheryl acetate, 70 mg; cholecalciferol, 6000 IU; menadione, 2.5 mg; riboflavin, 7 mg; thiamine, 4 mg; pantothenate, 12 mg; niacin, 50 mg; folate, 3 mg; pyridoxine, 6 mg; biotin, 300 μg; cyanocobalamine, 15 μg; Fe (sulfate), 30 mg; Cu (sulfate), 14 mg; Se (selenate), 0.3 mg; I (iodide), 1.20 mg; Zn (sulfate and oxide), 120 mg; Mn (sulfate and oxide), 100 mg.

### Virulent APEC O78 Challenge Model

Virulent field APEC O78 multidrug-resistant (MDR) strain used in the current experiment was previously recovered from broiler chickens with colisepticemia according to a previous research paper by one of the co-authors (35). The strain was cultivated onto macConkey's agar plates (Oxoid, UK) at 37°C for 24 h and the challenge inoculum was adjusted to give a final viable cell concentration of approximately 10^8^ CFU/ml (36). The virulence of the strain was verified by PCR investigation of the most important virulence genes; *papC, iroN, iutA*, and *iss* (37). Moreover, the strain was evidenced to be resistant to amoxicillin-clavulanic acid, aztreonam, amikacin, erythromycin, streptomycin, rifampicin, colistin, gentamycin, sulphamethoxazole/trimethoprim, ciprofloxacin, ceftriaxone, chloramphenicol, piperacillin, tetracycline, and imipenem being MDR. This resistance pattern was used as a re-isolation marker (38).

At 14 days of age, all birds in PC and eugenol nanoemulsion groups were orally inoculated, *via* crop gavage, with 0.5 ml of 3 × 10^8^ CFU/ml APEC O78 broth culture for experimental induction of colisepticemia, while NC group was kept unchallenged. The bacterial infection was ascertained *via* observing the characteristic clinical picture and gross lesions associated with colisepticemia besides re-isolation and identification of the infecting APEC O78 strain. Additionally, a successful establishment of the colisepticemia model was checked *via* re-examining the antimicrobial susceptibility profiles and the existence of the investigated virulence genes in the APEC O78 strain.

### Monitoring the Bird's Growth Performance

The feed intake (FI) and body weight (BW) were recorded for determination of the feed conversion ratio (FCR) and body weight gain (BWG) at the end of starter, grower, and finisher periods as described previously (10). The cumulative FI, BWG, and FCR were calculated accordingly over the whole rearing period (days 1–35). Mortality rates were recorded throughout the experimental period.

### Sampling

Blood samples were aseptically collected from the bird's wing vein and divided into two parts. The first part was kept with heparin for some hematological assays and the second part was collected in a clot activator vacutainer tube to allow clotting and centrifuged for 10 min at 3,000 rpm for separation of serum, which was kept at −20°C for further biochemical assays. Five birds/replicates were slaughtered and sacrificed. The cecal contents were aseptically removed from each bird, placed in sterile tubes, and frozen at −80°C for subsequent bacterial population counts *via* quantitative real-time PCR (qPCR) and for analysis of mRNA expression of APEC virulence genes. The pancreatic tissues were collected aseptically for subsequent gene expression analysis of digestive enzymes. Moreover, the jejunal segments were washed with sterile phosphate-buffered saline, cut aseptically about 30–35 cm proximally from mid-jejunum (Meckel's diverticulum), and subjected to expression analysis of barrier functions and cytokine related genes *via* reverse transcription-quantitative polymerase chain reaction (RT-qPCR) technique. Finally, the liver samples were used for re-isolation of challenge APEC O78 strain, which was subjected for TEM examination.

### Biochemical and Hematological Investigations

At 14 days (before challenge), the red blood cells (RBCs) were estimated *via* a Neubauer hemocytometer (Sigma-Aldrich, Germany) and hemoglobin (Hb) concentrations were calculated using the cyanomethemoglobin colorimeteric procedure. Moreover, the levels of alanine transaminase (ALT), aspartate transaminase (AST), total proteins, creatinine, uric acid, total cholesterol, triglycerides, and a diverse fraction of lipoproteins [low-density lipoprotein (LDL), very low-density lipoprotein (VLDL), and high-density lipoprotein (HDL)] were assessed using an automated spectrophotometer (Chemray 240. USSR). At 21 and 35 days of age (1st and 3rd weeks post-APEC O78 challenge, respectively), the levels of ALT, AST, total proteins, creatinine, and uric acid and activities of lysozyme (LYZ), myeloperoxidase (MPO), and nitric oxide (NO) were assessed by commerce kits (Jiancheng Biotechnology Institute™, Nanjing, China).

### Gene Expression Profile Analysis by RT-QPCR

At 14 and 35 days of age, pancreatic and jejunal tissues were used for determining the mRNA expression levels of genes encoding digestive enzymes [alpha 2A amylase (AMY2A), cholecystokinin (CCK), pancreatic lipase (PNLIP), and chymotrypsin-like elastase family, member 1 (CELA1)], barrier functions [cathelicidins-2, β-defensin-1, mucin-2 (MUC-2), junctional adhesion molecule-2 (JAM-2), occludin, claudins-1 (CLDN-1), and fatty acid-binding protein-2 (FABP-2)] and cytokines [interleukin-6 (IL-6), IL-8, IL-1β, IL-10, and tumor necrosis factor-alpha (TNF-α)]. Moreover, cecal contents were used for subsequent analysis of mRNA expression levels of APEC O78 virulence genes (*papC, iroN, iutA*, and *iss*) at the 1st and 3rd week post-APEC O78 challenge. RNA was separated by QIAamp RNeasy Mini kit (Qiagen, Hilden, Germany) as endorsed by the manufacturers' instructions. The RNA concentration was estimated at 260 nm and the RNA clarity was spectrophotometrically measured by computing the ratio of absorbance wave length at 260 and 280 nm. One-step RT-qPCR assays were achieved on the Strata-gene MX3005P real-time PCR recognition system by a QuantiTect SYBR Green RT-PCR Kit (Qiagen, Hilden, Germany). All PCR procedures were performed in triplicate. The specificity of all PCR amplifications was verified by a melting curve analysis. The transcripts expression levels were normalized to those of TATA-binding protein (TBP), glyceraldehyde 3-phosphate dehydrogenase (GAPDH), and *E. coli 16S rRNA* genes as endogenous controls. The gene-specific primer sequences exploited in RT-qPCR assay are presented in Table 2. The outcomes of relative mRNA expression of studied genes were assessed *via* the 2^−ΔΔCt^ method (42).

**Table 2:** Primer sequences used for quantitative PCR assays.

**Encoding gene**	**Primer sequence (5′-3′)**	**Accession No./** **Reference**
**Digestive enzymes**		
*AMY2A*	F: CGGAGTGGATGTTAACGACTGG R: ATGTTCGCAGACCCAGTCATTG	NM_001001473.2
*PNLIP*	F: GCATCTGGGAAG^↓^GAACTAGGG R: TGAACCACAAGCATAGCCCA	NM_001277382.1
*CCK*	F: AGGTTCCACTGGGAGGTTCT R: CGCCTGCTGTTCTTTAGGAG	XM_015281332.1
*CELA1*	F: AGCGTAAGGAAATGGGGTGG R: GTGGAGACCCCATGCAAGTC	XM_015300368.1
**Barrier functions**		
Cathelicidins-2	F: AGGAGAATGGGGTCATCAGG R: GGATCTTTCTCAGGAAGCGG	NM_001024830.3
β-defensin-1	F: AAACCATTGTCAGCCCTGTG R: TTCCTAGAGCCTGGGAGGAT	NM_204993.1
Occludin	F: ACGGCAAAGCCAACATCTAC R:ATCCGCCACGTTCTTCAC	XM_031604121.1
*CLDN-1*	F: GGTGAAGAAGATGCGGATGG R: TCTGGTGTTAACGGGTGTGA	NM_001013611
*MUC-2*	F: AAACAACGGCCATGTTTCAT R: GTGTGACACTGGTGTGCTGA	NM_001318434
*JAM-2*	F: AGACAGGAACAGGCAGTGCT R: TCCAATCCCATTTGAGGCTA	XM_031556661.1
*FABP-2*	F: AGGCTCTTGGAACCTGGAAG R: CTTGGCTTCAACTCCTTCGT	NM_001007923
**Cytokines**		
*IL-6*	F: AGGACGAGATGTGCAAGAAGTTC R: TTGGGCAGGTTGAGGTTGTT	NM_204628.1
*IL-8*	F: CTGGCCCTCCTCCTGGTT R: GCAGCTCATTCCCCATCTTTAC	XM_015281283.2
*IL-10*	F: GCTGAGGGTGAAGTTTGAGG R: AGACTGGCAGCCAAAGGTC	XM_025143715.1
*IL-1β*	F:GCTCTACATGTCGTGTGTGATGAG R: 50-TGTCGATGTCCCGCATGA	NM_204524
*TNF-α*	F: CGTTTGGGAGTGGGCTTTAA R: GCTGATGGCAGAGGCAGAA	NM_204267.1
**House keeping**		
*GAPDH*	F: CAACCCCCAATGTCTCTGTT R: TCAGCAGCAGCCTTCACTAC	NM205518
*TBP*	F: GTCCACGGTGAATCTTGGTT R: GCGCAGTAGTACGTGGTTCTC	Acc:8484
***E. coli***		
*16S rRNA*	F: GACCTCGGTTTAGTTCACAGA R: CACACGCTGACGCTGACCA	(39)
**Total bacteria**		
*16S rRNA*	F: CGGYCCAGACTCCTACGGG R: TTACCGCGGCTGCTGGCAC	(40)
***Enterobacteriaceae***		
*16S rRNA*	F: CATTGACGTTACCCGCAGAAGAAGC R: CTCTACGAGACTCAAGCT TGC	
***Bacteroides*** **species**		
*16S rRNA*	F: GAGAGGAAGGTCCCCCAC R: CGCTACTTGGCTGGTTCAG	
***Lactobacillus*** **species**		
*16S rRNA*	F: CACCGCTACACATGGAG R: AGCAGTAGGGAATCTTCCA	
***E. coli*** **O78 virulence**		
*papC*	F: GACGGCTGTACTGCAGGGTGTGGCG R: ATATCCTTTCTGCAGGGATGCAATA	(41)
*iroN*	F: AATCCGGCAAAGAGACGAACCGCCT R: GTTCGGGCAACCCCTGCTTTGACTTT	
*iutA*	F: GGCTGGACATCATGGGAACTGG R: CGTCGGGAACGGGTAGAATCG	
*iss*	F: CAGCAACCCGAACCACTTGATG R: AGCATTGCCAGAGCGGCAGAA	

*AMY2A, alpha 2A amylase; PNLIP, pancreatic lipase; CCK, cholecystokinin; CELA1, chymotrypsin-like elastase family, member 1; CLDN-1, claudins-1; MUC-2, mucin-2; JAM-2, junctional adhesion molecule-2; FABP-2, fatty acid binding protein-2; IL, interleukin; TNF-α, tumor necrosis factor alpha; GAPDH, glyceraldehyde-3- phosphate dehydrogenase; TBP, TATA-binding protein*.

### Quantitative Microbial Profiling

At 14, 21, and 35 days of age, DNA from frozen cecal digesta samples was extracted with QIAamp DNA Stool Mini Kit (Qiagen, Germany) following the manufacturer's instructions. The extracted DNA was subjected to qPCR assays for quantification of genomic DNA copies of total bacteria and some intestinal bacterial species, including *Lactobacillus, Enterobacteriaceae*, and *Bacteroids*, at 14 and 35 days of age and APEC O78 challenge strain at 1st and 3rd weeks post-APEC O78 challenge, in triplicate, using Stratagene MX3005P RT-PCR machine and SYBR Green PCR Master Mix (Qiagen, Germany) according to the manufacturer's protocol. The sequences of the primers targeting the bacterial-specific *16S rRNA* genes are shown in Table 2. The DNA samples extracted from pure bacterial cultures were 10-fold serially diluted to create the standard calibration curves. The number of target genomic DNA copies was calculated and the bacterial quantities were expressed in terms of log_10_ CFU per gram of the cecal digesta.

### Transmission Electron Microscopy

At 35 days of age, liver samples of 3 birds in all groups were collected for re-isolation of the challenge APEC O78 strain, which was subjected to TEM examination for detecting any morphological alterations. The re-isolated *E. coli* strain was cultivated onto MacConkey's agar plates at 37°C and then fresh pure colonies from agar plates were incubated at 37°C to the mid-logarithmic phase. The samples were prepared following the procedures of Bozzola and Russell (43) with modifications. Briefly, the bacterial specimens were fixed at room temperature at 1:1 v/v in 2% glutaraldehyde and 1% paraformaldehyde for 1 h. Afterward, the samples were dehydrated by a graded ethanol level (30, 50, 70, 90, and 100%). The specimens were then kept in pure propylene and transmitted to epoxy embedding resin. Uranyl acetate and lead citrate were used for staining of sample sections, which were investigated *via* a JEOL JEM 1010 TEM (Jeol Ltd., Tokyo, Japan) at the Regional Center for Mycology and Biotechnology, Al-Azhar University, Cairo, Egypt.

### Statistical Analysis

All obtained statistical data were analyzed *via* the GLM procedure of SPSS version 22. The homogeneity among the treatment groups was carried out through Levene's test and normality by Shapiro–Wilk's test using the model Y ik = μ + Li + eik, where Y ik is the observation, μ is the overall means, Li is the effect of experimental groups and eik is random error. Variations among the data were determined as SEM and the significance was indicated at *P* ≤ 0.05. Tukey's test was utilized to assess the significant differences among the mean values. All graphs were generated *via* the GraphPad Prism software Version 8.

## Results

### Growth Performance Parameters

Growth performance parameters before and in response to experimental APEC O78 infection are shown in Table 3. Significant differences were detected among different experimental groups along all rearing periods. In the starter period (days 1 to 10), BWG and FCR were prominently improved in groups supplemented with 250 and 400 mg/kg diet of eugenol nanoemulsion. Moreover, the FI was significantly stimulated (*P* < 0.05) by increasing the level of eugenol nanoemulsion. In the grower period (days 11 to 20), the *E. coli* challenge significantly (*P* < 0.05) reduced FI and BWG and increased FCR and survivability in the PC group compared to groups that received eugenol nanoemulsion and were challenged with *E. coli*, especially at higher doses. In the finisher period (days 21 to 35), supplementation of eugenol nanoemulsion significantly (*P* < 0.05) improved BWG and FCR, unlike the PC group. In an overall trial period (days 0 to 35), BWG, FI, FCR, and survivability were negatively affected by *E. coli* challenge in the group unsupplemented with eugenol nanoemulsion; nevertheless, its supplementation significantly (*P* < 0.05) boosted BWG and FCR of birds. Moreover, birds that received 400 mg/kg of eugenol nanoemulsion showed a higher significant survivability rate (96%) compared with the PC group (64%).

**Table 3:** Effects of dietary supplementation of various levels of eugenol nanoemulsion on growth performance parameters of broilers challenged with *E. coli* O78 at 14 days of age.

	**NC**	**PC**	**Eugenol nanoemulsion** **(mg/kg diet)**			***P*-value**	**SEM**
			**100**	**250**	**400**		
**Starter (1–10 days)**							
BW (g/bird)	312^d^	318^c^	319^c^	332^b^	341^a^	<0.001	5.91
BWG (g/bird)	267^d^	272^c^	274^c^	286^b^	295^a^	<0.001	5.29
FCR	1.29^a^	1.29^a^	1.29^a^	1.25^b^	1.20^a^	<0.001	3.8
FI (g/bird)	346^c^	352^bc^	354^ab^	359^a^	354^ab^	<0.001	7.41
**Grower (11–20 days)**							
BW (g/bird)	1236^e^	1087^d^	1211^c^	1299^b^	1322^a^	<0.001	8.6
BWG (g/bird)	924^e^	769^d^	892^c^	967^b^	981^a^	<0.001	9.4
FCR	1.64^bc^	2.00^a^	1.70^b^	1.65^bc^	1.61^c^	<0.001	<0.001
FI (g/bird)	1517^c^	1534^bc^	1516^c^	1594^a^	1578^ab^	<0.001	3.91
**Finisher (21–35 days)**							
BW (g/bird)	2479^b^	1996^d^	2245^c^	2481^b^	2635^a^	<0.001	3.2
BWG (g/bird)	1243^b^	909^d^	1034^c^	1182^b^	1313^a^	<0.001	9.2
FCR	1.68^e^	2.53^a^	1.97^b^	1.84^c^	1.74^d^	<0.001	<0.001
FI (g/bird)	2092^c^	2299^a^	2038^d^	2178^b^	2288^a^	<0.001	4.90
**Allover (1–35 days)**							
BWG (g/bird)	2434^b^	1951^d^	2199^c^	2435^b^	2589^a^	<0.001	3.38
FI (g/bird)	3954^c^	4184^ab^	3909^c^	4131^b^	4221^a^	<0.001	5.5
FCR	1.62^d^	2.15^a^	1.78^b^	1.70^c^	1.63^d^	<0.001	<0.001
Survivability (%)	96^a^	64^c^	86^b^	90^a^	96^a^	<0.001	1.5

*BW, body weight; BWG, body weight gain; FI, feed intake; FCR, feed conversion ratio; NC (negative control), birds fed on basal diet; PC (positive control), birds fed on basal diet and challenged with E. coli at day 14 of age; SEM, standard error of the mean. Means with different superscripts within the same row differ significantly (p <0.05)*.

### Biochemical and Hematological Analyses

At 14 days of age, there were no significant differences (*P* > 0.05) in the levels of ALT, AST, creatinine, and uric acid among all experimental groups. The concentration of serum total proteins was significantly (*P* < 0.05) increased with increasing levels of dietary eugenol nanoemulsion. The RBCs count tended to be increased in response to eugenol nanoemulsion, while no significant differences were detected in Hb concentrations among various experimental groups. Inclusion of different levels of eugenol nanoemulsion significantly (*P* < 0.05) decreased the cortisol and triglycerides levels unlike the control group and the most lowering values were detected in broilers fed 400 mg/kg of eugenol nanoemulsion. In contrast, the serum VLDL and LDL levels were greatly (*P* < 0.05) reduced post supplementation of higher levels of eugenol nanoemulsion (Table 4).

**Table 4:** Effects of dietary supplementation of different doses of eugenol nanoemulsion on serum biochemical parameters of broilers prior to *E. coli* O78 challenge.

**Parameter**	**Control**	**Eugenol nanoemulsion (mg/kg diet)**				
		**100**	**250**	**400**	***P-*value**	**SEM**
Total protein (g/dl)	4.00^d^	4.36^c^	5.10^b^	5.87^a^	<0.001	0.03
ALT (U/L)	33.32	31.33	32.33	32.17	0.20	0.48
AST (U/L)	46.37	46.17	45.00	46.43	0.34	0.60
Uric acid (μmol/L)	9.92	10.06	9.83	10.03	0.89	0.10
Creatinine (mg/dl)	0.27	0.28	0.30	0.28	0.21	0.01
RBCs (×10^6^/μL)	2.42^b^	2.47^ab^	2.52^a^	2.48^ab^	0.05	0.01
Hb (g/dl)	12.27	12.17	12.20	12.23	0.95	0.13
Cholesterol (mg/dl)	124.03^a^	124.57^a^	113.73^b^	103.78^c^	<0.001	1.18
Triglycerides (mg/dl)	97.78^a^	85.43^b^	83.70^b^	73.70^c^	<0.001	4.06
HDL (mg/dl)	39.93	35.56	39.04	39.89	0.18	2.99
LDL (mg/dl)	65.55^b^	71.92^a^	57.95^c^	49.15^d^	<0.001	2.88
VLDL (mg/dl)	19.53^a^	17.09^b^	16.74^b^	14.74^c^	<0.001	0.16

*ALT, alanine transaminase; AST, aspartate transaminase; RBCs, red blood cells; Hb, hemoglobin; HDL, high-density lipoprotein; LDL, low-density lipoprotein; VLDL, very low-density lipoprotein; SEM, standard error of the mean. Means with different superscripts within the same row differ significantly (P < 0.05)*.

At the 1st and 3rd weeks post *E. coli* challenge, the unsupplemented and challenged birds exhibited the highest levels of ALT, AST, creatinine, and uric acid. Meanwhile, their levels were restored after dietary supplementation of eugenol nanoemulsion, especially at the dose of 400 mg/kg. Moreover, the highest level of serum total proteins was observed in birds fed 400 mg/kg of eugenol nanoemulsion at both intervals (Table 5). During 1st week post *E. coli* challenge, the inclusion of higher levels of eugenol nanoemulsion significantly (*P* < 0.05) reduced the LYZ activities unlike the PC group; meanwhile the group fed 400 mg/kg of eugenol nanoemulsion restored the LYZ activities to be similar to those in the NC group at 3rd-week post *E. coli* challenge. Notably, the serum activities of MPO and NO contents were significantly decreased (*P* < 0.05) in birds fed 250 and 400 mg/kg of eugenol nanoemulsion compared with the PC group at both time points (Table 6).

**Table 5:** Effects of dietary supplementation of various concentrations of eugenol nanoemulsion on serum biochemical parameters of broilers post-challenge with *E. coli* O78.

**Experimental group**		**1st** **week post challenge**					**3rd** **week post challenge**				
		**Total** **protein (g/dl)**	**ALT (U/L)**	**AST (U/L)**	**Uric acid (μmol/L)**	**Creatinine (mg/dl)**	**Total** **protein (g/dl)**	**ALT (U/L)**	**AST (U/L)**	**Uric acid (μmol/L)**	**Creatinine (mg/dl)**
Eugenol,	PC	1.48^e^	68.73^a^	82.83^a^	17.80^a^	0.62^a^	1.53^c^	54.97^a^	75.30^a^	16.53^a^	0.56^a^
(mg/kg)	NC	4.07^a^	33.24^e^	46.57^c^	10.30^d^	0.30^c^	4.10^a^	32.80^d^	46.50^c^	10.17^d^	0.31^cd^
	100	2.40^d^	55.20^b^	56.53^b^	16.97^a^	0.43^b^	3.37^b^	43.60^b^	52.17^b^	13.97^b^	0.37^b^
	250	2.87^c^	47.40^c^	56.30^b^	15.17^b^	0.37^bc^	3.73b	37.20^c^	47.83^c^	12.17^c^	0.35^bc^
	400	3.60^b^	38.83^d^	54.73^b^	12.57^c^	0.34^c^	4.20^a^	33.53^d^	46.43^c^	9.57^d^	0.29^d^
	*P*-value	<0.001	<0.001	0.03	<0.001	<0.001	0.02	<0.001	0.04	<0.001	<0.001
	SEM	0.31	0.68	0.08	0.06	0.00	0.01	0.02	0.02	0.02	0.09

*PC (positive control), birds fed basal diet and challenged with E. coli O78 at 14 days of age; NC (negative control), birds fed basal diet; SEM, standard error of the mean; ALT, alanine transaminase; AST, aspartate transaminase. Means with different superscripts within the same row differ significantly (P < 0.05)*.

**Table 6:** Effects of dietary supplementation of different levels of eugenol nanoemulsion on serum immunological parameters of broilers post challenge with *E. coli* O78.

**Experimental group**		**1st** **week post challenge**			**3rd** **week post challenge**		
		**LYZ (U/ml)**	**MPO (U/L)**	**NO (μmol/L)**	**LYZ (U/ml)**	**MPO (U/L)**	**NO** **(μmol/L)**
Eugenol (mg/kg)	PC	217.49^a^	47.47^a^	8.90^a^	148.80^a^	30.47^a^	9.40^a^
	NC	144.75^d^	33.50^c^	6.23^c^	104.72^d^	24.46^b^	3.88^d^
	100	171.47^b^	37.87^b^	7.13^b^	127.08^b^	31.63^a^	5.32^c^
	250	157.15^c^	32.97^c^	6.53^bc^	120.94^bc^	28.53^b^	4.68^cd^
	400	151.33^cd^	32.40^c^	6.33^c^	113.13^cd^	26.17^b^	3.35^d^
	*P*-value	<0.001	<0.001	0.03	0.04	<0.001	<0.001
	SEM	0.02	2.01	1.06	0.92	0.04	0.11

*PC (positive control), birds fed basal diet and challenged with E. coli O78 at 14 days of age; NC (negative control), birds fed basal diet; LYZ, lysozyme; MPO, myeloperoxidase; NO, nitric oxide. Means with different superscripts within the same row differ significantly (P < 0.05)*.

### Expression Analysis Data

**Figure 2:**
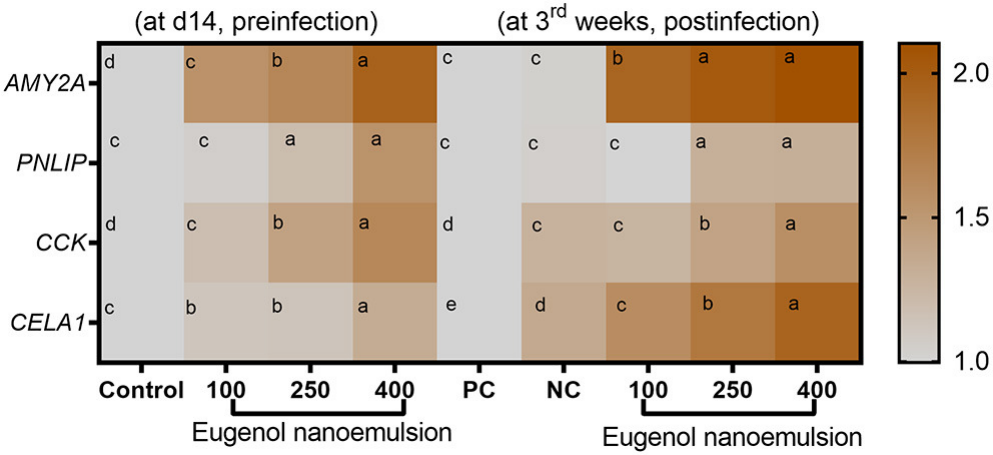
Heat map demonstrating the expression levels of genes related to digestive enzymes; AMY2A (alpha 2A amylase), PNLIP (pancreatic lipase, CCK (cholecystokinin), and CELA1 (chymotrypsin-like elastase family, member 1) in the pancreas of broiler chickens received different levels of eugenol nanoemulsion pre- (at day 14) and post- (at 3rd week) *E. coli* O78 challenge detected by RT-qPCR technique. Values are means with their SE. The intensity of orange color denotes the degree of upregulation of the investigated genes. NC (negative control): birds fed basal diet without eugenol nanoemulsion and were not challenged, PC (positive control): birds fed a control diet without eugenol nanoemulsion and were challenged, eugenol nanoemulsion 100, 250, and 400: birds fed basal diet supplemented with 100, 250, and 400 mg/kg diet eugenol nanoemulsion. All groups except NC were challenged with *E. coli* O78 at 14 days of age. Different letters within the same row indicate a statistical significance (*P* < 0.05).

**Figure 3:**
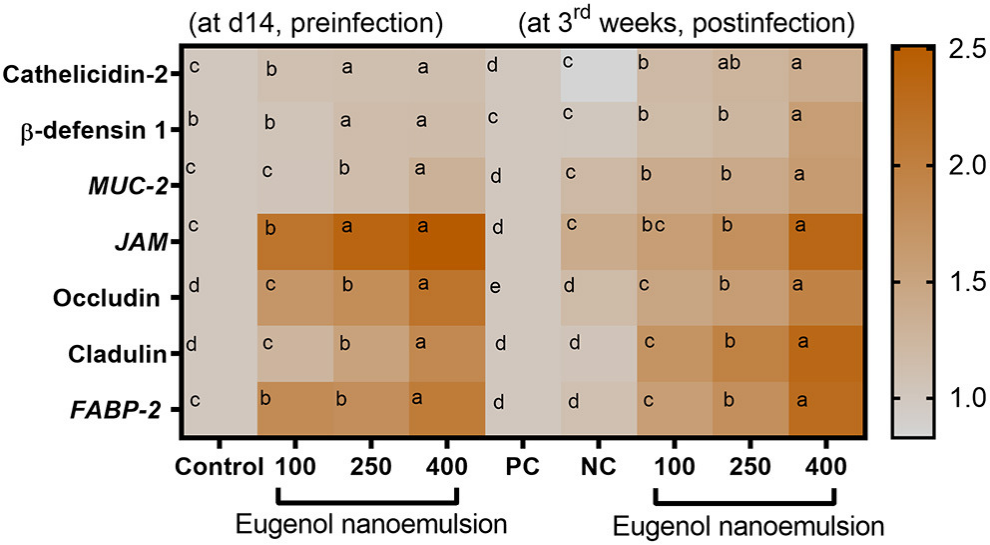
Heat map illustrating the gene expression profiles associated with barrier functions; cathelicidins-2, β-defensin-1, MUC-2 (mucin-2), JAM-2 (junctional adhesion molecule-2), occludin, CLDN-1 (claudins-1), and FABP-2 (fatty acid binding protein-2) in the jejunal tissues of broiler chickens supplemented with different levels of eugenol nanoemulsion pre- (at day 14) and post- (at 3rd week) *E. coli* O78 challenge determined by RT-qPCR assay. Values are means with their SE. The intensity of orange color denotes the degree of upregulation of the investigated genes. NC (negative control): birds fed basal diet without eugenol nanoemulsion and were not challenged, PC (positive control): birds fed a control diet without eugenol nanoemulsion and were challenged, eugenol nanoemulsion 100, 250, and 400: birds fed basal diet supplemented with 100, 250, and 400 mg/kg diet eugenol nanoemulsion. All groups except NC were challenged with *E. coli* O78 at 14 days of age. Different letters within the same row indicate a statistical significance (*P* < 0.05).

**Figure 4:**
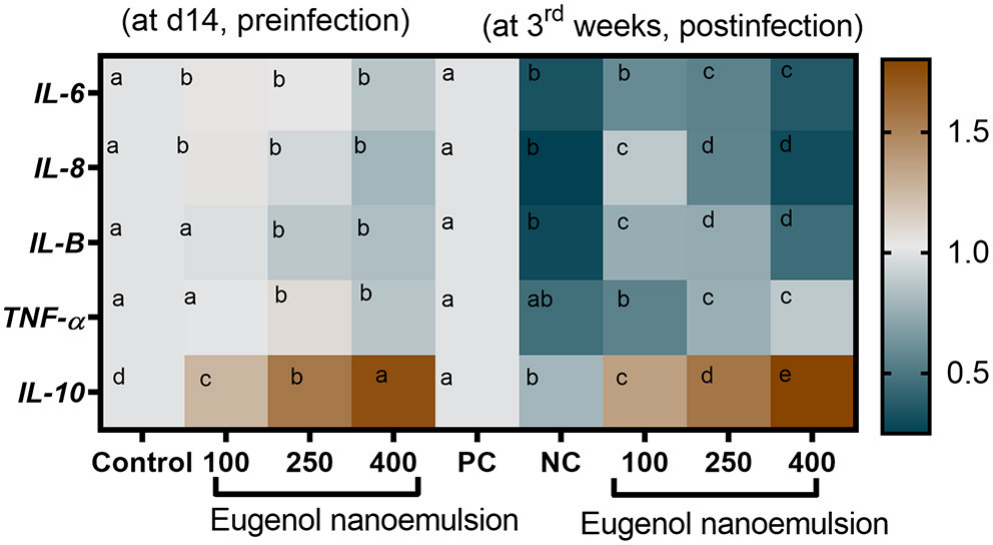
Heat map depicting RT-qPCR analysis of the relative expression levels of mRNAs encoding cytokines; IL-6 (interleukin-6), IL-8, IL-1β, IL-10, and TNF-α (tumor necrosis factor alpha) in the jejunal segments of broiler chickens fed different levels of eugenol nanoemulsion pre- (at day 14) and post- (at 3rd week) *E. coli* O78 challenge. Values are means with their SE. The intensity of orange and blue colors denotes the degree of upregulation and downregulation of the investigated genes, respectively. NC (negative control): birds fed basal diet without eugenol nanoemulsion and were not challenged, PC (positive control): birds fed a control diet without eugenol nanoemulsion and were challenged, eugenol nanoemulsion 100, 250, and 400: birds fed basal diet supplemented with 100, 250, and 400 mg/kg diet eugenol nanoemulsion. All groups except NC were challenged with *E. coli* O78 at 14 days of age. Different letters within the same row indicate a statistical significance (*P* < 0.05).

The expression levels of genes encoding digestive enzymes before and after *E. coli* challenge are illustrated in Figure 2. Before *E. coli* challenge, the transcription of *AMY2A* and *CCK* genes was significantly (*P* < 0.05) upregulated with increasing the levels of eugenol nanoemulsion compared with the control group. Moreover, the highest significant (*P* < 0.05) expression levels of *CELA1* and *PNLIP* genes were detected in the group supplemented with eugenol nanoemulsion at the level of 400 mg/kg. Interestingly, *AMY2A* and *PNLIP* transcriptional levels reached their peaks (*P* < 0.05) after dietary inclusion of 250 and 400 mg/kg eugenol nanoemulsion even after *E. coli* challenge. Furthermore, *CCK* and *CELA1* genes mRNA expression levels were significantly (*P* < 0.05) upregulated, especially with higher supplementation levels of eugenol nanoemulsion. The expression profiles of gene encoding barrier functions are presented in Figure 3. Prior to challenge, the transcription levels of β-defensin-1 and cathelicidins-2 genes were not statistically (*P* > 0.05) significant in response to dietary supplementation of eugenol nanoemulsion. Moreover, dietary supplementation of eugenol nanoemulsion at various levels significantly (*P* < 0.05) increased the transcriptional levels of genes encoding TJPs, including occludin and CLDN-1, in a dose-dependent manner unlike the control group. Of note, the highest prominent (*P* < 0.05) transcription level of *MUC-2* gene was observed in group supplemented with eugenol nanoemulsion at the level of 400 mg/kg (increased by 1.31-fold). Moreover, the expression of *JAM-2* gene was significantly increased in groups fed 250 and 400 mg/kg eugenol nanoemulsion groups (increased by 2.37 and 2.51-fold), followed by 100 mg/kg eugenol nanoemulsion group (2.16-fold increase). The expression level of *FABP2* gene was highly reported in groups fed 400 mg/kg eugenol nanoemulsion group (2.05-fold increase), followed by 250 and 100 mg/kg eugenol nanoemulsion groups (1.83- and 1.79-fold increase). Notably, *E. coli* challenge did not negatively alter the relative expression levels of barrier functions related genes upon dietary eugenol nanoemulsion supplementation as evidenced by their significant (*P* < 0.05) higher levels, especially with increasing the concentrations of dietary eugenol nanoemulsion. As shown in Figure 4, the quantitative expression of genes encoding cytokines was prominently affected either earlier to or after the *E. coli* challenge. Before the challenge, the group fed 400 mg/kg eugenol nanoemulsion exhibited the highest significant (*P* < 0.05) expression levels of *IL-6* and *IL-8* genes. Moreover, dietary supplementation of 250 and 400 mg/kg eugenol nanoemulsion significantly (*P* < 0.05) downregulated the mRNA expression levels of *IL-1*β and *TNF-*α genes in comparison with the control group. Regarding the *IL-10* relative expression levels, dietary inclusion of eugenol nanoemulsion significantly (*P* < 0.05) upregulated its levels in a dose dependent manner. Post *E. coli* challenge, dietary eugenol nanoemulsion supplementation significantly (*P* < 0.05) restored the excessive expression levels of *IL-6, IL-8, IL-1*β, and *TNF-*α genes to be nearly similar to those in the unchallenged group. Moreover, significant (*P* < 0.05) increased *IL-10* mRNA expression levels were observed in groups supplemented with eugenol nanoemulsion in a dose-proportional manner, unlike the PC group.

### Quantitative Profiling of Cecal Microbial Loads

**Figure 5:**
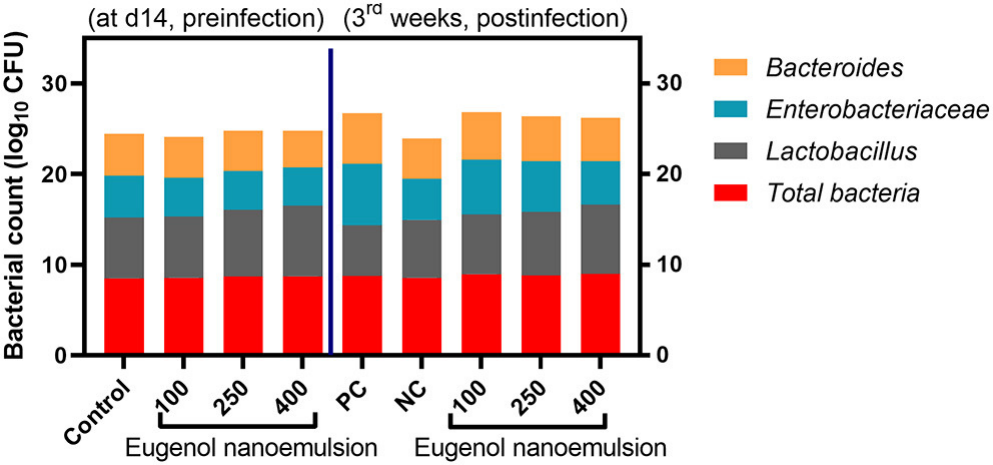
The impact of dietary inclusion of various levels of eugenol nanoemulsion on the populations (log_10_ CFU) of total bacteria, *Lactobacillus, Enterobacteriaceae*, and *Bacteriods* as was estimated by qPCR assay in the cecal digesta of broiler chickens pre- (at day 14) and post- (at 3rd week) *E. coli* O78 challenge. Values are means with their SE. NC (negative control): birds fed basal diet without eugenol nanoemulsion and were not challenged, PC (positive control): birds fed a control diet without eugenol nanoemulsion and were challenged, eugenol nanoemulsion 100, 250, and 400: birds fed basal diet supplemented with 100, 250, and 400 mg/kg diet eugenol nanoemulsion. All groups except NC were challenged with *E. coli* O78 at 14 days of age.

The profiling of cecal microbiota prior to and later to *E. coli* challenge in different experimental groups is depicted in Figure 5. There were no significant (*P* > 0.05) differences observed in the total bacterial loads in response to dietary various levels of eugenol nanoemulsion inclusion prior to *E. coli* challenge. *Bacteroides* counts exhibited no significant (*P* > 0.05) variations in all experimental groups except for the group fed 400 mg/kg eugenol nanoemulsion, where its loads decreased by 13% compared with the control group. Moreover, *Lactobacillus* counts were markedly (*P* < 0.05) increased in 250 and 400 mg/kg eugenol nanoemulsion supplemented groups in relation to the control group (7.4 and 7.8 vs. 6.7 log_10_ CFU/g, respectively). The most prominent (*P* < 0.05) reduction of *Enterobacteriaceae* counts was noticed in the group fed 400 mg/kg eugenol nanoemulsion. At 35 days of age (3rd-week post *E. coli* challenge), there was no noticeable effect (*P* > 0.05) for dietary eugenol nanoemulsion on total bacterial loads among all challenged groups. Moreover, *Bacteroides* and *Enterobacteriaceae* counts notably (*P* < 0.05) decreased with the increasing level of dietary eugenol nanoemulsion in challenged broilers, unlike the PC group. In contrast, birds supplemented with 400 mg/kg eugenol nanoemulsion retained considerably higher significant (*P* < 0.05) *Lactobacillus* counts even after *E. coli* challenge.

### Quantification of *E. coli* O78 DNA Copies

**Figure 6:**
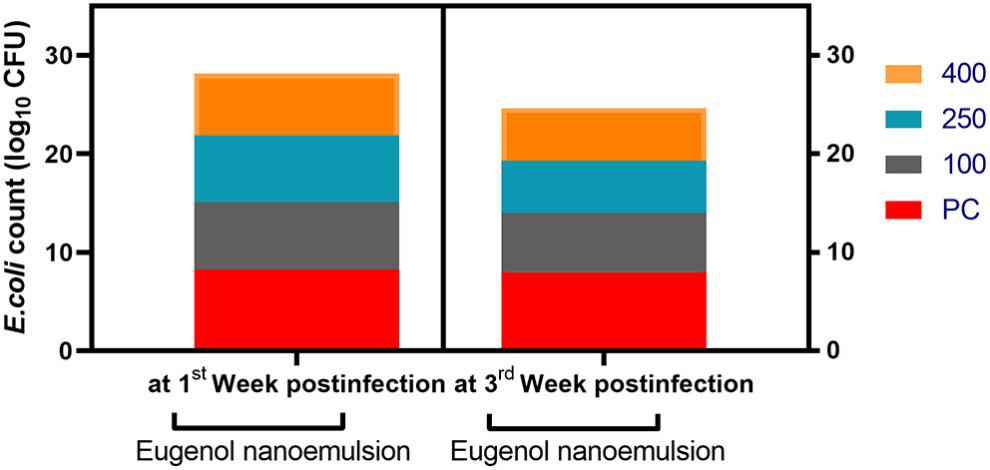
Quantification of cecal APEC O78 loads (log_10_ CFU) in response to eugenol nanoemulsion supplementation at the 1st and 3rd weeks post-challenge as was measured by qPCR assay. Values are means with their SE. NC (negative control): birds fed basal diet without eugenol nanoemulsion and were not challenged, PC (positive control): birds fed a control diet without eugenol nanoemulsion and were challenged, eugenol nanoemulsion 100, 250, and 400: birds fed basal diet supplemented with 100, 250, and 400 mg/kg diet eugenol nanoemulsion. All groups except NC were challenged with *E. coli* O78 at 14 days of age.

The results of quantification of *E. coli* O78 in the cecal digesta of broilers are described in Figure 6. At the 1st-week post-challenge, the lowest significant (*P* < 0.05) log_10_ copies of *E. coli* O78 populations were observed in the cecal contents of broilers fed 400 mg/kg eugenol nanoemulsion (1.91 log units decreases than the PC group). Another notable finding to emerge from our data was the significant (*P* < 0.05) reduction in loads of pathogenic *E. coli* O78 in groups supplemented with eugenol nanoemulsion unlike the PC group in a dose-dependent manner at the 3rd-week post *E. coli* challenge.

### Expression Analysis of *E. coli* Virulence Genes

**Figure 7:**
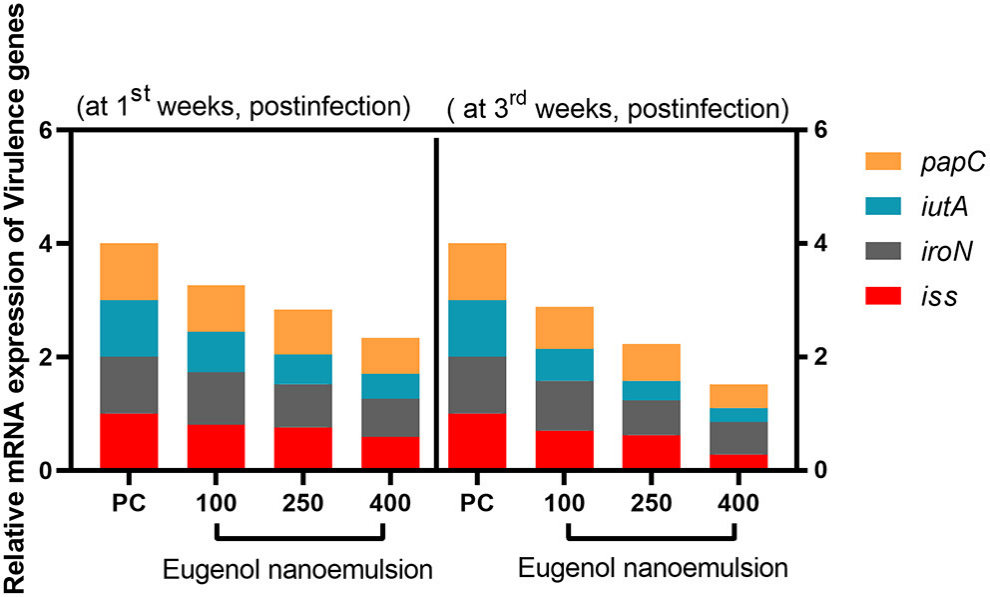
RT-qPCR investigation of the relative mRNA expression levels of *E. coli papC, iutA, iroN*, and *iss* virulence genes in the cecal contents of broiler chickens fed different levels of eugenol nanoemulsion at the 1st- and 3rd-week post-challenges. Values are means with their SE. NC (negative control): birds fed basal diet without eugenol nanoemulsion and were not challenged, PC (positive control): birds fed a control diet without eugenol nanoemulsion and were challenged, eugenol nanoemulsion 100, 250, and 400: birds fed basal diet supplemented with 100, 250, and 400 mg/kg diet eugenol nanoemulsion. All groups except NC were challenged with *E. coli* O78 at 14 days of age.

Data displayed in Figure 7 showed the expression levels of *E. coli papC, iroN, iutA*, and *iss* virulence genes post supplementation of eugenol nanoemulsion. The most marked reduction (*P* < 0.05) in *iss* and *papC* mRNA expression levels was detected in the group that received eugenol nanoemulsion at the concentration of 400 mg/kg, followed by 100 and 250 mg/kg at the 1st and 3rd week post *E. coli* challenge. The relative mRNA expression levels of *E. coli iroN* gene were significantly (*P* < 0.05) downregulated in broilers that received eugenol nanoemulsion, especially at higher levels at both time points. Moreover, eugenol nanoemulsion inclusion significantly (*P* < 0.05) reduced the *iutA* gene expressions at both time intervals (down to 0.44- and 0.25-fold, respectively in 400 mg/kg supplemented group compared with the PC group).

### Transmission Electron Microscopy Analysis of *E. coli*

Transmission electron microscopy of the re-isolated *E. coli* strains from the liver samples of broiler chickens in the PC group showed that the bacterial cells retained their normal bacillary shape and flagella with intact cell wall and uniform cytoplasm (Supplementary Figure 1a). The *E. coli* strains recovered from the broiler chickens in the eugenol nanoemulsion supplemented groups revealed signs of cell wall deformation, shriveling in the cells with loss of their structure, and the cells did not retain much of their rod shape indicating bacterial cell damage as revealed by TEM (Supplementary Figures 1b–d). This adverse effect was most prominent post supplementing eugenol nanoemulsion higher levels.

## Discussion

Avian pathogenic *E*. *coli* often causes a decline in the immune defenses leading to significant economic fatalities. Using of antimicrobials greatly enhanced the animals yield (44); but, these settlements have been compromised by the expansion of microbial resistance (45–47).

In the existing study, dietary supplementation of eugenol nanoemulsion was capable of modulating broiler chickens' immune responses to overcome experimental *E. coli* infection and targeting their maximal potential growth performance. Herein, increasing the level of eugenol nanoemulsion had improved the overall growth rate and FCR earlier to experimental *E. coli* infection (stater period). Meanwhile, the loss of body weight and impaired FCR post-infection in the PC group, a result of an inflammatory process and intestinal damage, were restored in the group supplemented with eugenol nanoemulsion at the level of 400 mg/kg diet. In agreement with our results, birds fed different levels of EOs over the period of 1–42 days showed increased BWG and better FCR when compared with the control unsupplemented group (48). Based on previous studies, the positive impact of EOs on the growth performance of broiler chickens could be attributed to their bioactive compounds capacity in augmenting the digestive and pancreatic enzymes secretion (48) and enhancing immune functions (49). Moreover, stimulating appetite and antimicrobial properties are likely to be other reasons explaining the roles of EOs in enhancing growth performance (11). Nevertheless, the mode of action of phytogenic extracts is yet to be defined and it may differ due to various sources, forms, and compositions of active elements being used in diets. Notably, growth performance parameters of broilers chickens fed thymol and carvacrol EOs were not impaired after *Clostridium perfringens* (*C. perfringens*) challenge (8). Similarly, dietary supplementation of nano encapsulated cumin essential oil at the level of 200 mg/kg diet improved the FCR of broiler chickens (50). Moreover, a recent study described the growth-promoting effect of a mixture of eugenol and garlic tincture in birds challenged with *C. perfringens* (22); however, the efficiency of eugenol in its nano form on broilers performance was not be investigated until now.

Interestingly, the better growth rate and feed utilization in eugenol nanoemulsion supplemented groups came in alignment with increasing the expression of genes encoding digestive enzymes (AMY2A, CCK, PNLIP, and CELA1). It has been stated that phytogenic extracts and their active principles can control the expression profiles of digestive genes in ileal mucosa (51) and stimulate the digestive secretions for enhancing nutrients digestibility (52). The possible mechanisms of EOs on growth performance could be due to the higher feed digestibility by triggering the endogenous enzymes and regulating the GIT microbial flora (53). Moreover, broilers fed dietary EOs had augmented lipase, trypsin, and chymotrypsin secretions (54). It was previously proven that EOs had positive impacts on the nutrient digestibility of broiler chickens (22). However, upregulating the digestive enzyme gene expression following eugenol nanoemulsion was not studied until now. Its boosting role on broilers performance could be clarified by potentiating the bioavailability and bioactivity of eugenol as eugenol nanoemulsion permits a deeper tissue penetration and simplifies cellular uptake in the GIT, which leads to well-organized upregulation of the digestive enzymes' genes. Besides, better broiler's performance following dietary supplementation of eugenol nanoemulsion even after exposure to *E. coli* infection could be attributed to its optimistic role in modulating the immune response and lowering the infection severity without prompting antimicrobial resistance.

During the starter period, the existing data prior to the challenge revealed non-significant changes on liver and kidney function tests using various levels of eugenol nanoemulsion explaining that eugenol nanoemulsion did not have any negative impact on liver and kidney functions at such levels (55). Where there was an evidence for the hypolipidemic activity of eugenol nanoemulsion at the levels of 250 and 400 mg/kg with more pronounced effects for the high dose, it was suggested that the cholesterol-reducing impact induced by eugenol nanoemulsion was entirely attributed to LDL reduction in Harb et al. (55). Later to *E. coli* challenge, liver and kidney functions showed a rise in ALT, AST, uric acid, and creatinine activities, which could be due to the harmful effects of bacterial infection on hepato-renal tissues (56, 57). However, supplementation of eugenol nanoemulsion, especially at the level of 400 mg/kg revealed a gradual decline of these parameters toward the normal levels as those in the NC group concluding its hepato-renal protective effects (58). Bacterial infection persuades systemic inflammatory reactions, which is a critical problem in inducing stress on immune functions that threaten the health status and result in impaired bird performance (59). Phagocytic cells are mainly responsible for the production of NO, LYZ, and MPO (60). Increased NO and LYZ levels and MPO activities could be a response to bacterial challenge stimulation and are the vital indicators of inflammatory reactions (61). Meanwhile, lessening NO along with LYZ and MPO levels at the 1st and 3rd week post-infection in groups supplemented with various levels of eugenol nanoemulsion, with more pronounced effects for the high dose, revealed its potential role in alleviating the harmful effects of bacteria. This may be related to the anti-inflammatory action of dietary eugenol nanoemulsion (62, 63).

The intestinal mucosa is not only the main site for nutrient digestion and absorption, but it also plays a crucial role in the host defense against pathogens and prevents the leakage of proinflammatory molecules *via* the intestinal mucosa to the circulatory system (64). Integrity of intestinal mucosa is maintained by tight junctions (65) those are critical for establishment of an intact physical barrier among the intestinal epithelial cells (66). The disruption of TJPs is a major cause of the “leaky guts” that could lead to reduction in the nutrient absorption, elevation in the permeability to luminal antigens, bacterial translocation, tissue damage, and sustained inflammation (67). Supplementation of phytogenic active principles into nanoforms and assessing their impacts on broiler intestinal barrier integrity are considered new issues that require more investigations to understand their mode of actions. The focus on eugenol nanoemulsion prophylactic roles for protecting broilers against *E. coli* infection has not been investigated until now. In this regard, our findings described that broiler chickens fed eugenol nanoemulsion, especially at higher levels considerably upregulated the expression of genes encoding TJPs (occludin, claudins-1, and JAM-2) suggesting its role in strengthening the barrier integrity before infection as well as restoring its function even after *E. coli* experimental infection. In agreement with our results, higher gene expression levels of TJPs and augmented intestinal barrier function were noticed upon thymol nanoemulsion supplementation, especially at higher concentrations (68). Moreover, thymol and carvacrol (69) have been shown to enhance the expression of genes involved in barrier functions in broilers infected with *C. perfringens*. Administration of phytogenic extracts exhibited a profound impact on the expression of genes encoding TJPs resulting in higher levels of Zona occludin at ileal and cecal levels as well as CLDN1, CLDN5, and OCLN at the cecal level (68). Phytogenics could enhance intestinal barrier integrity *via* promoting the assembly of TJPs (70) that may lead to higher protection against toxic feed substances or endogenously formed toxic metabolites (71). Additionally, mucin is produced from goblet cells and secreted into the intestinal lumen forming a protective layer, which protects the gut from acidic chyme, digestive enzymes, and pathogens (50, 72). Besides TJPs, the mucus layer is the first defense barrier faced by intestinal bacteria, where mucins are the main components of the mucus layer (73). Remarkably, mucin implies the first line of immune defense and augmenting its release is helpful in inhibiting the invasion of pathogens and spread of toxins into the GIT (74). The inflammatory lesions diminish the mucin secreted from goblet cells, prevent mucosal layer regeneration and trigger further infection, bacterial translocation, and intestinal inflammation (9, 75). Interestingly, increased expression levels of *MUC-2* and *FABP-2* genes were detected following dietary eugenol nanoemulsion administration. In accordance, it has been shown that birds fed a mixture of microencapsulated eugenol and garlic EOs had increased intestinal integrity and enhanced mucin-secreting goblet cells (40), which can further prove their protective impacts against necrotic enteritis. Moreover, FABP harmonize cells lipid responses and are recognized as major contributions to both inflammatory and metabolic pathways (76). Regarding *E. coli* infection, the lower expression levels of *MUC-2* and *FABP-2* genes in infected and untreated birds were associated with excessive gut inflammation. The reduction of *MUC-2* in challenged animals would be due to lowering the capacity for mucosal renewal (77). In contrast, their higher expression levels in birds supplemented with higher concentrations of eugenol nanoemulsion and infected with *E. coli* is an evidence of the gut barrier recovery and dysbacteriosis (78). Similarly, birds fed dietary EOs (clove, *Artemisia sieberi, Coriondrum sativum L*, and *Myrtus communis*) upregulated the expression of *MUC-2* gene in the jejunum unlike the control treatment (79). The bioactive substances may modify the activity of transcription factors that regulate mucin-2 gene expression in broiler chickens (80). Besides its protective functions, mucin has a role in nutrients filtration in the GIT and it also can affect the nutrients digestion and absorption (81), which can explain the better growth performance of broiler chickens in this study. Recently, a mixture of microencapsulated eugenol and garlic EOs had increased the expression levels of *CLDN-1* and *JAM-2* genes in birds challenged with *C. perfringens* unlike the infected and untreated birds (40).

Host defense peptides (HDP) are broad-spectrum antimicrobial molecules those are expressed by the intestinal mucosa and take part in the intestinal innate immunity and mucosal defense and their gene expression depend on the microbial modulation (82–84). The main function of HDP is their antimicrobial activity and they also engaged in other roles, including chemotaxis, immunomodulation, or wound repair (85). Firm immunological barriers between the host and the intestinal antigens can be achieved through the binding of intestinal HDP with mucins (83). Moreover, a relation between intestinal HDP gene expression and microbiota composition, such as *E. coli*, was found (86). Additionally, a recent emerging evidence has highlighted the beneficial effects of HDP on mucosal barrier permeability by direct regulation of mucin and TJPs genes‘ expression (83). The defensins are cystine-rich antimicrobial peptides with broad antimicrobial activities as they can trigger macrophages and immature dendritic cells to reach mucosal tissues *via* chemokine receptors and also boost specific immunity against pathogenic bacteria (87). Moreover, defensins could act directly on bacterial pathogens and more importantly play a vital role in the innate immunity helping in adaptive immune response initiation and regulation (88). Cathelicidins are produced by epithelial and mucosal cells and leucocytes, where they are preserved in specific granules (89) and display broad antimicrobial activities mediated *via* direct interaction with and disruption of the microbial cell membrane (90). Remarkably, the cathelicidins and defensins synergistic activity implies their combined role in the orchestration of the innate host defense (91). Consistent with the above-mentioned facts, we demonstrated that the expression of HDP genes, including β-defensin-1 and cathelicidins-2, was prominently increased post supplementation of eugenol nanoemulsion, especially at higher levels, indicating its effective role in stimulating the mucosal defense; this finding was in the same line with the high expression levels of TJPs related genes.

Cytokines play important regulatory roles in the intestinal inflammatory response. During bacterial invasion into the intestinal epithelial cells, gastrointestinal immune cells are triggered to secrete cytokines, which play potential roles in the immune responses against pathogens (92). TNF-α and IL-1β are the important proinflammatory cytokines, which regulate the host immune response against many pathogens through differentiation and proliferation of the immune cells, NO production, and apoptosis (93). Meanwhile, over and long-term secretion of proinflammatory cytokines may cause gut damage (94). Intestinal IL1-β is expressed in the cells of lamina propria during intestinal health as well as disease conditions and its low expression yields positive effects in the intestinal mucosa and faster epithelia healing in case of inflammation (95). In addition, IL-6, IL-8, and TNF-α are initiating an inflammatory response by recruiting antimicrobial cells, such as neutrophils and macrophages (93). In the current study, increased intestinal gene expression levels of proinflammatory cytokines (TNF-α, IL-1β, IL-6, and IL-8) were observed in broilers fed a control diet and experimentally challenged with *E. coli*. As anticipated in our study, the administration of eugenol nanoemulsion had the most pronounced regulatory effects on the intestinal gene expression of proinflammatory cytokines as evidenced by suppressing their expression levels that might counteract inflammation caused by *E. coli* and therefore improve gut health. This can be attributed to the role of dietary eugenol nanoemulsion in enhancing the non-specific immunity in the body by nonspecific killing of fungi, bacteria, tumor cells, and parasites and thereby decreasing the pathogenic loads as was previously interpreted (96). On the other hand, IL-10 has predominantly opposing and complex roles in inflammation and it plays a vital role in suppressing the inflammatory and immune responses (97). Herein, the upregulated *IL-10* gene in groups that received dietary eugenol nanoemulsion, especially at higher levels can suppress the excessive inflammation and maintain the intestinal immune homeostasis indicating its strong anti-inflammatory properties. In accordance, 60 mg/kg of a blend of EOs exhibited anti-inflammatory properties by reducing the expression of the *TNF-*α gene expression and increasing that of *IL-10* gene in the broilers challenged with lipopolysaccharides injections (8) considering their regulatory roles. The appropriate immune response induced in our study after supplementing broiler chickens with eugenol nanoemulsion, especially at higher levels, which was evidenced by reduction of the expression of proinflammatory cytokines and enhancing that of the anti-inflammatory one could be due to the uniformly dispersed nanodroplets of eugenol nanoemulsion with more effective anti-inflammatory activities (98).

The intestinal microbiome plays a considerable role in reinforcing and maintaining the intestinal epithelial barriers and the immune system, which is critical for host protection against pathogenic microorganisms. In the current study, dietary eugenol nanoemulsion altered the cecal microbial composition of total bacteria, *Enterobacteriaceae, Lactobacillus*, and *Bacteroides* species before and after *E. coli* challenge. The intestinal microbiome is comprised of a huge number of symbiotic bacterial species that benefit the host by inhibiting the colonization of pathogenic bacteria *via* many mechanisms, such as augmentation of the immune responses, direct killing of the pathogens, and competitive exclusion (99). The findings observed in the present study revealed that broiler chickens fed higher levels of eugenol nanoemulsion had increased (*P* < 0.05) beneficial *Lactobacillus* counts in the cecal contents compared with the control groups suggesting the positive selection of eugenol nanoemulsion toward *Lactobacillus* species. In agreement with our study, Mohammadi et al., reported higher (*P* < 0.05) *Lactobacillus* counts in the caecum of broiler chickens after dietary inclusion of clove essential oil (100). Moreover, Agostini et al. revealed that clove stimulates the *Lactobacillus* proliferation in broiler chickens (101). Recently, higher ileal *Lactobacillus* loads were reported in broiler chickens fed a microencapsulated product consisted of garlic and eugenol tincture (40). *Bacteroides* species loads are increased in the intestine of birds when they are infected with bacterial pathogens (102). Herein, the increased loads of *Bacteroides* species in the intestine were associated with higher *E. coli* loads post-challenge. These bacteria exhibited excessive proteolytic and immunostimulatory activities, which impair the immune response and negatively affect intestinal health (103). Our results demonstrated that broiler chickens supplemented with higher levels of eugenol nanoemulsion had reduced populations of cecal *Bacteroides* species compared with the control groups. Moreover, *Enterobacteriaceae* counts followed the same decreasing trend following eugenol nanoemulsion dietary inclusion indicating its valuable effects against pathogenic bacterial species. Similarly reduced loads of *Bacteroides* species were observed in birds fed a microencapsulated product comprised of garlic and eugenol tincture (40). Moreover, feeding chickens on a product of plant EOs containing active components, such as eugenol decreased the abundance of cecal *Enterobacteriaceae* (104). The selective microbial effect of eugenol nanoemulsion proved in our study against pathogenic bacteria could hypothesize its promising *in vivo* antimicrobial properties. This may be linked to the fact that nanoemulsions offer a wide surface area, so they could permit the active components to penetrate faster and directly damage the bacterial membranes (68, 105).

Although *E. coli* is considered a commensal bacterium in the GIT of birds, some pathogenic serovars can invade various tissues and cause diseases, such as colisepticemia, enteritis, and colibacillosis (106). In this regard, APEC serotype O78 has been associated with the majority of infectious diseases worldwide with remarkable economic losses in the poultry industry (106). In the present study, quantitative analysis of cecal *E. coli* post-challenge revealed that dietary supplementation of eugenol nanoemulsion, especially at higher levels significantly (*P* < 0.05) decreased *E. coli* loads at the 1st-week post-challenge with respect to the PC group. Moreover, *E. coli* populations were greatly (*P* < 0.05) affected by dietary eugenol nanoemulsion inclusion in a dose-dependent manner at the 3rd-week post-challenge. This pronounced reduction in *E. coli* counts corroborated data from previous investigators, where the density of ileal *E. coli* was decreased (*P* < 0.05) after supplementing broiler chickens with a trademark essential oil mixture containing eugenol (22). Moreover, clove essential oil decreased (*P* < 0.01) *E. coli* counts in the caecum of broiler chickens in comparison with the control group indicating its inhibitory effects against *E. coli* (100).

Transmission electron microscopy of *E. coli* strains re-isolated from the liver samples of broiler chickens supplemented with eugenol nanoemulsion revealed reduction in the size of cells, cells shrinkage with signs of deformation in the cell wall unlike the normal features observed in *E. coli* strains re-isolated from the control group. Other investigators have corroborated our observations; Di Pasqua et al. (107) revealed similar results supporting the morphological alterations described for *E. coli* cells treated with eugenol using a scanning electron microscope confirming its promising antimicrobial activity against *E. coli*. Although the precise mechanism of action of phytogenic-derived molecules on bacteria is not obviously understood, many hypotheses have been suggested to elucidate the mode of their antimicrobial activities, which varies among such products‘ sources. Nonetheless, their general anticipated mechanisms of action are *via* disintegrating the bacterial pH gradient and affecting the bacterial cell permeability (11, 108). A critical enhanced antimicrobial property of eugenol or its nanoformulations is owing to its hydrophobicity (109), which helps in targeting the mitochondria and lipid-containing bacterial cell membranes, destroying membrane integrity and subsequently leading to leakage of intracellular substances (25, 26). Other possible mechanisms of action beyond their antimicrobial effects are related to damaging membrane proteins, depleting the proton motive force, and coagulating the bacterial cytoplasm (26). Herein, it seems reasonable that eugenol nanoemulsion stimulated the intestinal production of mucus in broiler chickens with consequent impairment of *E. coli* adhesion as was previously documented (11). Targeting bacterial virulence using new anti-virulence therapies is considered a potential alternative approach that can be used to disarm bacterial pathogens (2). From this point of view, our results revealed that the beneficial effect of eugenol nanoemulsion on *E. coli* was then ascertained by its downregulating effect on *E. coli papC, iutA, iroN*, and *iss* virulence genes. Previous studies are continuously recording the *in vitro* modulatory effect of eugenol on the expression of virulence genes of *E. coli* serovars (110, 111). However, to the best of our knowledge, there are no data reporting the *in vivo* effect of eugenol nanoemulsion on APEC O78 virulence in broiler chickens. The outcome of this finding clearly proved the enhanced survivability of broiler chickens challenged with *E. coli* upon supplementation with eugenol nanoemulsion compared with the unsupplemented control birds offering new insights on its mechanism of action and suggesting a powerful tool to control colisepticemia induced by APEC in broilers.

## Conclusion

Formulation of eugenol into the nanoemulsion form efficiently controlled and sustained its release in GIT and thus boosted its bioavailability, growth-promoting efficiency, and antimicrobial activity against APEC O78. These beneficial effects were more prominent after dietary inclusion of eugenol nanoemulsion at the level of 400 mg/kg and could arise from its promising role in accelerating the digestive enzyme gene expression and preserving the gut barrier functions. Finally, the cross-talk between underlying multifocal mechanisms of eugenol nanoemulsion and adaptive responses in broiler chickens could provide new insights for controlling colisepticemia induced by APEC and in turn target the goal of maximum production in poultry farming.

## Data Availability Statement

The datasets presented in this study can be found in online repositories. The names of the repository/repositories and accession number(s) can be found in the article/Supplementary Materials.

## Ethics Statement

The experiment procedures were approved by university strategies for the care of experimental animals and they have been certified by the ZU-IACUC Board of the Faculty of Veterinary Medicine, Zagazig University, Egypt.

## Author Contributions

DI, FE, AM, EA-A, DM, TI, TH, GA, AN-A, and MA contributed to conceptualization, methodology, software, validation, formal analysis, investigation of resources, reviewing and editing the manuscript, visualization, supervision, and funding. DI, TI, TH, and MA contributed to data curation and writing the original draft. All authors contributed to the article and approved the submitted version.

## Conflict of Interest

The authors declare that the research was conducted in the absence of any commercial or financial relationships that could be construed as a potential conflict of interest.

## Publisher's Note

All claims expressed in this article are solely those of the authors and do not necessarily represent those of their affiliated organizations, or those of the publisher, the editors and the reviewers. Any product that may be evaluated in this article, or claim that may be made by its manufacturer, is not guaranteed or endorsed by the publisher.
