# Supplementary figures and images for: Dietary Eugenol Nanoemulsion Potentiated Performance of Broiler Chickens: Orchestration of Digestive Enzymes, Intestinal Barrier Functions and Cytokines Related Gene Expression With a Consequence of Attenuating the Severity of E. coli O78 Infection
Source: Front Vet Sci. 2022 Jun 23;9:847580. doi: 10.3389/fvets.2022.847580 (PMC9260043; doi:10.3389/fvets.2022.847580)

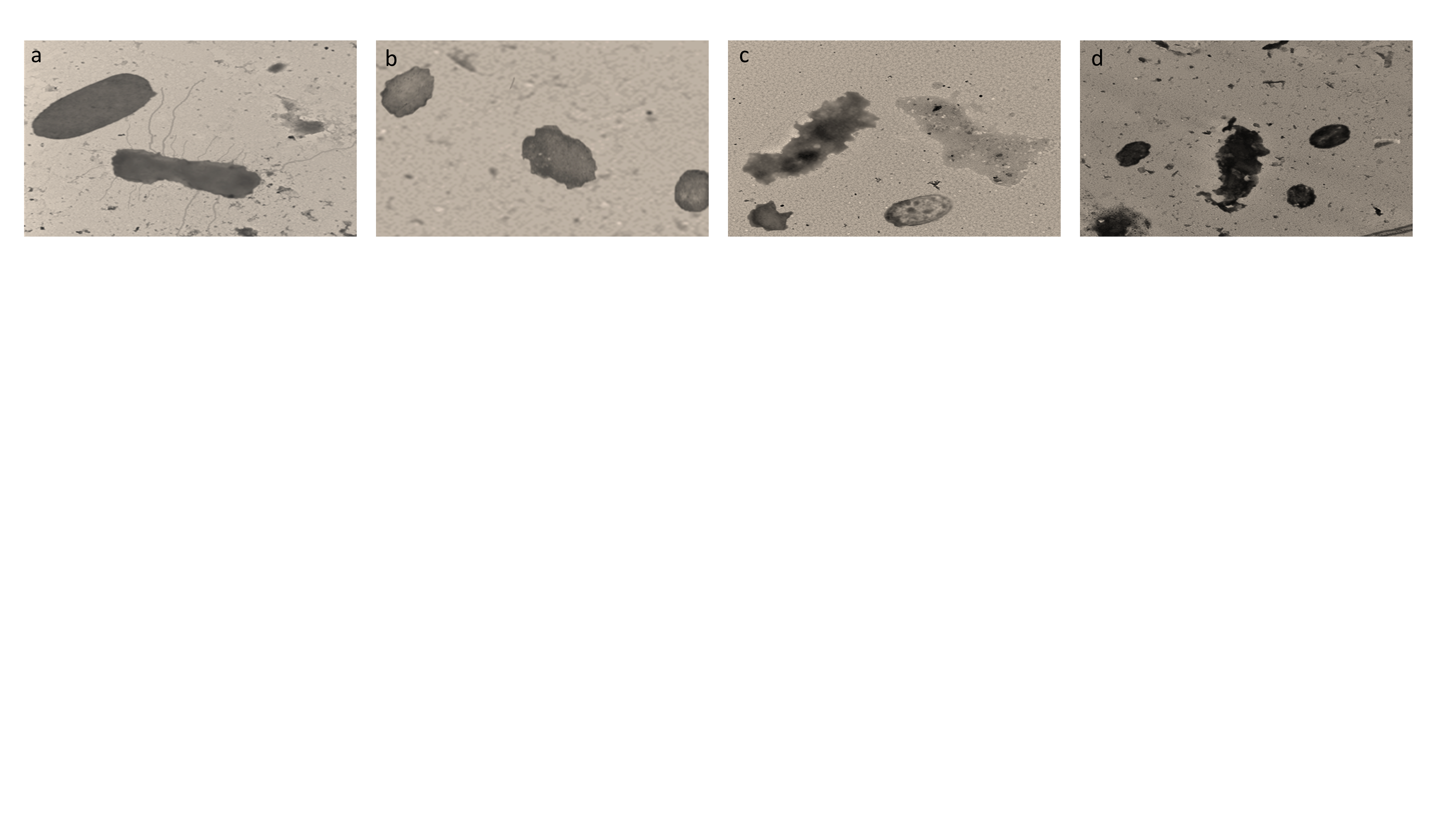

Supplement: Supplementary file 1 [file Image_1.TIF]
